# Surgical training for simple and complex hernia repair in the UK: results of a nationwide training survey

**DOI:** 10.1308/rcsann.2025.0065

**Published:** 2025-10-01

**Authors:** R Willmott, SG Parker, D Slade, S Halligan, D Sanders, DR Clyde, L Smith, P Daliya, JD Hodgkinson, T Badenoch, D Damaskos, O Ali, J Torkington, R Thomas

**Affiliations:** ^1^ University College London Hospitals NHS Foundation Trust, UK; ^2^ Croydon University Hospital, UK; ^3^ Salford Royal Hospitals NHS Foundation Trust, UK; ^4^ University College London Centre of Medical Imaging, UK; ^5^ Royal Devon University Healthcare NHS Foundation Trust, UK; ^6^ Western General Hospital, UK; ^7^ University Hospital of Wales, UK; ^8^ Sherwood Forest Hospitals NHS Foundation Trust, UK; ^9^ Royal Berkshire NHS Foundation Trust, UK; ^10^ Gloucester Royal Hospital, UK; ^11^ Royal Infirmary of Edinburgh/NHS Lothian, UK; ^12^ Gateshead NHS Foundation Trust, UK

**Keywords:** Hernia, Abdominal wall, General surgery, Curriculum, Specialties, Surgical

## Abstract

**Introduction:**

Abdominal wall reconstruction (AWR) is increasingly recognised as a subspecialty in general surgery, owing to the growing complexity and advancement of hernia repair techniques. Concerns have been raised among UK hernia specialists about current surgical training adequately preparing trainees for both simple and complex hernia procedures.

**Methods:**

A CHERRIES-compliant survey was developed by a panel of hernia experts to evaluate UK training in hernia surgery. The 41-item questionnaire assessed perceived competence and confidence in performing eight types of hernia repair, categorised as simple (primary inguinal, umbilical, laparoscopic inguinal and Rives–Stoppa) or complex (recurrent inguinal, component separation and parastomal hernia repair), along with broader AWR-related topics (open abdomen management, participation in multidisciplinary meetings). The survey was disseminated via social media, targeted chat groups and surgical conferences.

**Results:**

The survey was conducted from 21 January to 27 September 2024. Of approximately 500 possible respondents, 116 completed the survey (47 surgical trainees (ST) 7–8s, 30 clinical fellows and 34 consultants), yielding an estimated 22.2% response rate. Curriculum requirements were met only for open inguinal and umbilical hernia repair. Although there are no formal curriculum requirements for complex repairs, trainee exposure remains limited; two-thirds had performed fewer than ten recurrent inguinal or component separation procedures. For parastomal hernias, confidence was highest with suture repair despite these being associated with poor outcomes. Overall, median confidence scores were highest for simple repairs and lowest for complex ones.

**Conclusions:**

Current UK surgical training provides inadequate exposure to complex AWR, highlighting the need for targeted curriculum improvement.

## Introduction

Hernia repair is one of the commonest surgical procedures in the UK, with over 120,000 performed annually.^1^ It encompasses a spectrum of procedures, from simple primary repairs to complex recurrent procedures. Despite hernia operations being among the most frequently performed in general surgery, outcomes vary significantly, influenced strongly by the surgeon’s expertise and operative experience. In the UK, there are now calls for subspecialisation in hernia surgery and training from consultant surgeons.^2,3^ In the USA and Europe this specialist interest has been termed abdominal wall reconstruction (AWR).

The growing complexity of hernia repair or AWR has raised concerns among hernia specialists regarding the readiness of new consultants to perform such surgery competently and independently. A study from the USA found that general surgery residents were ‘not universally ready’ to perform even core procedures, such as inguinal hernia repair, by the time they had completed their residency.^4^ With abdominal wall hernias increasing in prevalence as populations become increasingly obese and recurrence rates after repair remaining concerningly high, the adequacy of surgical training has become a critical area of investigation.

In the UK, training standards for hernia surgery are guided by curriculum-based competencies; however, explicit procedural benchmarks, indicative procedure numbers and recognition of newer AWR techniques are absent. The effectiveness of current training structures in preparing surgeons for varying degrees of procedural complexity has not been assessed comprehensively.

This study aims to evaluate the adequacy of training among senior surgical trainees and early years consultants across the whole range of AWR from simple to complex hernia repair, as well as broader AWR-associated topics such as management of the open abdomen and attendance at an AWR multidisciplinary team (MDT) meeting. By systematically examining trainees’ experience and confidence levels across a range of hernia repair techniques, we seek to identify specific areas requiring curricular revision and improved training standards to improve surgical proficiency and patient outcomes.

## Methods

To assess the competency and confidence of general surgical trainees in hernia surgery at the end of their training, we designed an open survey. The survey was developed by a team of hernia surgery experts via a series of online meetings and in accordance with CHERRIES (Checklist for Reporting Results of Internet E-Surveys) guidelines.^5^ The current curriculum assesses hernia repair competency at completion of surgical trainee year six (ST6). Therefore, we surveyed ST7 and ST8 trainees, as well as clinical fellows, and newly appointed consultants (both clinical fellows and consultants had to be within two years of completing their Certification of Completion of Training (CCT)). Trainees and young consultants from all general surgery subspecialties were included.

After discussing the survey with trainee programme directors, we estimated that approximately 500 UK surgeons (senior trainees, clinical fellows and young consultants) would be eligible to complete the survey.

The survey aimed to evaluate proficiency in performing simple and complex hernia repairs, including open primary/recurrent inguinal, open umbilical, laparoscopic transabdominal preperitoneal/total extraperitoneal (TAPP/TEP) inguinal, primary/recurrent incisional, posterior component separation (transversus abdominis release (TAR)), anterior component separation (external oblique release) and parastomal hernia repair. The survey was piloted on general surgery trainees at a regional AWR teaching day at Croydon University Hospital on Monday 4 September 2023 and refined following feedback. Supplementary Material 1 displays the final version.

The survey comprised 41 questions, structured around 3 recurring questions for each procedure as follows:

1. At what level are you currently performing this procedure? (options: not seen/not trained in this; assisting; supervisor trained scrubbed; supervisor unscrubbed; performed; I’d rather not say).
2. How many procedures have you performed during training? (free text)
3. How comfortable will you be/are you at performing this procedure as a consultant?

This was rated on a ten-point Likert scale,^6^ and used to assess confidence and ability in the same way as previous trainee surveys in the USA.^7^

Additional questions in some sections included which procedures respondents had been taught and their preferred techniques. Concluding questions explored respondents’ experience in more complex but related topics such as managing the open abdomen and their attendance at abdominal wall MDT meetings.

Respondents who answered ‘not seen/not trained in this’ for a particular procedure were instructed to skip subsequent questions in that section. If they continued to answer, their responses were excluded from the final analysis. For some of the more advanced techniques where respondents indicated they were only ‘assisting’ the procedure, this number was reported, and their confidence-level responses were excluded from analysis. The survey was distributed using the online survey platform Microsoft Forms (MS Forms updated June 2023, Microsoft, Redmond, WA, USA).

Questionnaire completion was voluntary but incentivised by entry into a ballot for free registration to the British Hernia Society’s conference in November 2024, and participation in a robotics hernia course. Before participation, respondents were provided with a detailed statement regarding the study purpose, ethical approval, and data retention and disposal protocols (in accordance with GDPR legislation). Participants were then required to confirm they understood this statement before participation, consenting for their responses to be collated and analysed. This study was approved by the Research and Development department at Croydon University Hospital.

The survey was initially disseminated via email to trainees by contacting training programme directors and trainee representatives at the Royal College of Surgeons England and of Edinburgh. This was followed by informal dissemination via social media, deanery-wide mobile phone group chats, mobile phone society group chats and personal contacts. Further dissemination was achieved face-to-face at conferences using hand-held tablets. Authors SGP, RW, DS and LS attended ACPGBI in Newport, Wales, 1–3 July 2024, and JDH and PD attended ASGBI in Belfast 8–10 May 2024. Our response rate was calculated by taking the total number of respondents and dividing by the estimated number of eligible UK respondents.

## Results

**Figure 1:**
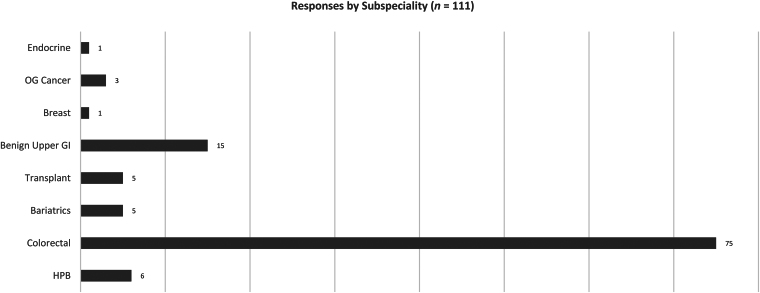
Designated subspecialty of respondents GI = gastrointestinal; HPB = hepato-pancreato-biliary; OG = oesophago-gastric

The survey was conducted from 21 January to 27 September 2024 inclusive. There were 116 responses out of an estimated 500 surgeons contacted, of which 5 were duplicates (and the second response excluded), leaving 111 for analysis (estimated response rate of 22.2%). Respondents included 24 ST7 trainees, 23 ST8 trainees, 30 clinical fellows and 34 consultant surgeons. There was representation from eight general surgical subspecialties (Figure 1). One respondent completed to question 32 only, with their responses to this point included in the analysis. The average response time was 8min 56s (excluding four outliers, whose completion times exceeded 60min).

**Figure 2:**
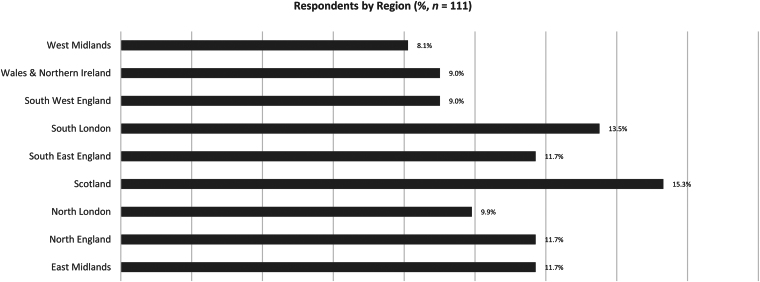
Respondents’ region of training

A total of 109 (98.2%) respondents stated they were intending to perform, or were performing, hernia operations as part of their consultant practice; 13 (11.7%) respondents did not consent to their answers being validated against their logbook. A total of 75 (67.5%) respondents were trained in colorectal surgery, and 15 (13.5%) were upper gastrointestinal surgeons. There were six or fewer respondents for the remaining general surgery subspecialties (Figure 1). Responses originated from all UK regions (Figure 2). The results for each type of hernia repair are described below, with the corresponding data broken down by average number performed by each grade in Tables 1–7. We have presented our results in order of increasing hernia repair complexity, beginning with simple open hernia repair procedures, so that readers can clearly see the changes in confidence score and number performed. Supplementary material 2 shows our final question results.

**Table 1:** Average number of procedures performed

	Simple				Complex		
	Primary open inguinal hernia repair	Open umbilical hernia repair	Laparoscopic TAPP/TEP inguinal hernia repair	Rives-Stoppa incisional hernia repair	Recurrent open inguinal hernia repair	Posterior component separation	Anterior component separation
Average number performed across all grades (range)	99 (10–500)	56 (6–501)	21 (0–90)	13 (1–75)	13 (1–200)	7 (1–50)	4 (1–25)
Average number performed by grade							
ST7	79	44	10	9	9	6	3
ST8	84	42	18	12	10	5	2
Clinical fellowship	92	51	27	19	10	9	5
Consultant surgeon (within 2 years of CCT)	128	78	26	13	21	6	5

CCT = certification of completion of training; ST = surgical trainee; TAPP = transabdominal preperitoneal; TEP =  total extraperitoneal

**Table 2:** Median confidence scores procedure

	Simple				Complex		
	Primary open inguinal hernia repair	Open umbilical hernia repair	Laparoscopic TAPP/TEP inguinal hernia repair	Rives-Stoppa incisional hernia repair	Recurrent open inguinal hernia repair	Posterior component separation	Anterior component separation
Median confidence score (IQR)	10 (9–10)	10 (9–10)	6 (4–8)	7 (5–9)	8 (6–9)	6 (5–7)	5.5 (4–7)
Median confidence score by grade							
ST7	9	9	5	5.5	7	4	4
ST8	10	9	5	7	7	5	5
Clinical fellowship	9	10	7	7	8	7	6.5
Consultant surgeon (within 2 years of CCT)	10	10	7	8	8	6	6

CCT = certification of completion of training; IQR = interquartile range; ST = surgical trainee; TAPP = transabdominal preperitoneal; TEP =  total extraperitoneal

**Table 3:** Parastomal hernia repair (complex) – number performed and confidence score

	Average number performed (range)	Median confidence scores
Across all grades	7 (0–21)	6
By grade		
ST7	5	4
ST8	7	5
Clinical fellowship	8	7
Consultant surgeon (within 2 years of CCT)	7	7

CCT = certification of completion of training; ST = surgical trainee

**Table 4:** Taught and preferred methods of parastomal hernia repair

Method of repair	Methods taught/learnt during training (frequency)	Preferred method (%)
Suture repair	91	49 (46.7%)
Sugarbaker	53	5 (4.8%)
Laparoscopic Sugarbaker	35	17 (16.2%)
Keyhole repair	28	8 (7.6%)
Laparoscopic keyhole repair	18	4 (3.8%)
Open Pauli parastomal repair/TAR release	16	4 (3.8%)
Hybrid repair with 3D funnel mesh	14	7 (6.7%)
I'd rather not say	2	11 (10.5%)
*n*=	257	105

TAR = transversus abdominis release

**Table 5:** Preferred plane for mesh re-enforcement in incisional hernia repair

Plane	All responses (%)
Onlay	3 (2.7%)
Inlay	4 (3.6%)
Retrorectus	87 (79.1%)
Intraperitoneal	2 (1.8%)
Preperitoneal	13 (11.8%)
I do not perform incisional hernia repair	0 (0.0%)
I'd rather not say	1 (0.9%)
*n*=	110

**Table 6:** Preferred technique to manage open abdomen

Technique	All responses (%)
I have not seen this/I do not perform this	2 (1.8%)
Commercial NPWT (AbThera)	70 (64.2%)
Homemade NPWT (Barker or Sandwich VacPac)	6 (5.5%)
Bogota bag	4 (3.7%)
Deep tension sutures	5 (4.6%)
NPWT and mesh-mediated traction	20 (18.3%)
I'd rather not say	2 (1.8%)
*n*=	109

NPWT = negative pressure wound therapy

**Table 7:** Involvement (observed or taken part in/contributed) in abdominal wall MDT

MDT Involvement	Number of responses	Response
Never taken part in one	52	47.3%
Local AWR MDT	33	30.0%
Regional AWR MDT	20	18.2%
I'd rather not say	5	4.5%
*n*=	110	

AWR = abdominal wall reconstruction; MDT = multidisciplinary team

### Simple hernia repair techniques

#### Primary open inguinal hernia repair

Of 108 respondents, 70 (64.8%) had performed between 50 and 125 primary open inguinal hernia repairs, with the number increasing with seniority of respondent (as expected). Confidence performing this procedure as a consultant was high, with 98 (90.8%) respondents rating their comfort level at 8 or higher (median 10). Out of 34 consultants, 19 (55.9%) rated their comfort level 10.

#### Open umbilical hernia repair

Of 103 respondents, 65 (63.1%) had performed between 26 and 75 open umbilical hernia repairs. Confidence was high, with median confidence score for all grades being 10.

#### Laparoscopic TAPP/TEP inguinal hernia repair

Out of 90 respondents, 75 (83.3%) had performed 30 or fewer laparoscopic inguinal hernia repairs, with a mean number performed of 21 (range, 0–90). Respondents’ median confidence was low, however, with a median score of 6, with some respondents rating their confidence as 1 or 2 for performing this procedure. Preferred technique for laparoscopic inguinal hernia repair was split, with 53 (58.9%) respondents preferring TAPP repair and 37 (41.1%) preferring TEP repair.

#### Rives–Stoppa incisional hernia repair

Out of 90 respondents, 55 (61.1%) had performed ten or fewer Rives–Stoppa incisional hernia repairs, with ST7 trainees comprising the largest individual group (81.3%). Only two (2%) respondents had performed more than 40 procedures, with the mean number performed across all grades being 13. The median confidence score for a primary incisional hernia repair was 7, while the score for repairing a recurrent incisional umbilical hernia was 6. Confidence in both procedures increased with seniority.

### Complex hernia repair techniques

#### Recurrent open inguinal hernia repair

Of 101 respondents, 73 (72.3%) had performed between 1 and 10 recurrent open inguinal hernia repairs, with a mean of 13 (range, 1–200). Four (4%) had performed the procedure more than 30 times. Confidence performing this procedure as a consultant was variable, with scores ranging from 3 to 10 (median 8), but with nine (31%) consultants rating their confidence level as 10.

#### Posterior component separation (or transversus abdominis release) and anterior component separation (external oblique release)

For posterior component separation, 39 of 56 (69.6%) respondents had performed five or fewer, and for anterior component separation, 55 of 64 (85.9%) had performed five or fewer. The mean number of posterior component separations performed was 7 (range, 1–50) compared with 4 (range, 1–25) for anterior component separation. No ST7 or ST8 trainee had performed more than five anterior component separation procedures, whereas three (56%) had performed more than five posterior component separations. These findings were reflected in confidence scores, with a median score of 6 for posterior component separation and 5.5 for anterior component separation. Clinical fellows reported the highest confidence scores for both procedures; 7 for posterior component separation and 6.5 for anterior component separation.

#### Parastomal hernia repair

For parastomal hernia repair, we asked respondents to select which techniques they had been taught during training. Of 257 responses, 91 (35.4%) selected suture repair; 53 (21%) open Sugarbaker repair; 35 (14%) laparoscopic Sugarbaker repair; 28 (10.9%) keyhole repair; 18 (7%) laparoscopic keyhole repair; 16 (6%) the open Pauli technique; 14 (5%) the hybrid funnel technique; and 2 (1%) selected ‘I’d rather not say’. Of these techniques, almost half of respondents (49 of 105, 46.7%) were most comfortable with suture repair, and this was consistent across all grades. Two-thirds (51 of 79; 64.6%) of respondents had performed 5 or fewer repairs, with a mean of 7 (range, 0–23). Median confidence score was 6, with a noticeable difference among grades; 4 for ST7 trainees versus 7 for consultants.

### Incisional hernia repair/MDTs/open abdomens

The majority of respondents, 87 of 110 (79.1%), reported retrorectus as their preferred plane for mesh reinforcement for incisional hernia repair. This finding was consistent among all grades: 18/24 (75%) ST7s; 18/23 (78.3%) ST8s; 25/29 (86.2%) clinical fellows; 26/34 (76.5%) consultants. For management of open abdomens, 70 of 109 (64.2%) respondents preferred commercial negative pressure wound therapy (NPWT) dressings. Of 109 respondents, 20 (18.3%) selected NPWT with mesh-mediated fascial traction, which was the second most-selected option. Nearly half of respondents had never observed or taken part in an abdominal wall MDT (52/110, 47.3%), and only 1 in 5 (20/110, 18.2%) reported attending a regional AWR MDT.

## Discussion

Our study highlights concerns regarding the adequacy of training in both simple and complex hernia surgery in the UK. With an increasing prevalence of abdominal wall hernias presenting to the general surgery clinic,^8^ and notable recurrence rates post-repair,^9^ there is a pressing need for more structured training.

Our survey has indicated that, in inguinal hernia, trainees typically met current curriculum requirements for simple primary open repair, but experience with more complex recurrent inguinal hernia and simple laparoscopic techniques was inadequate. Most senior trainees completed fewer than 30 laparoscopic repairs, and fewer than 10 recurrent open inguinal repairs, markedly below the known established learning curves of 50–100 procedures.^10,11^

Training gaps were even more pronounced for ventral hernia repair. Encouragingly, the majority of respondents selected the retro-rectus plane as their preferred plane of mesh insertion; however, most respondents reported performing fewer than ten primary incisional hernia (Rives–Stoppa) procedures, considered the most basic of midline repairs. Respondents also reported performing fewer than five complex procedures such as anterior or posterior component separation. In parastomal hernia, which is complex and a source of significant morbidity and mortality, trainees and early years consultants reported fewer than ten procedures throughout their entire training. Given evidence linking volume to reductions in complications and recurrence rates, it is reasonable to conclude that insufficient training could adversely affect patient outcomes.^12,13^

Overall confidence levels among respondents correlated inversely with procedural complexity, with senior trainees rating themselves 5 or below for anterior and posterior component separation. The authors note that respondents were slightly more confident in performing posterior component separation compared with anterior component separation and had performed more of these. Considering posterior component separation is much harder to perform and less commonly performed, we suspect there is a Dunning–Kruger effect^14^ here, i.e. respondents may have a poor understanding of posterior component separation and were overrating numbers and confidence scores. In addition, respondents reported higher confidence scores for parastomal hernia repair; however, this reflected suboptimal techniques such as suture repair (much easier to perform), which is universally discouraged due to its inevitable failure,^15^ and is indicated only in emergency settings.^16^ These findings underscore the discrepancy between perceived confidence and expertise, indicating a significant training deficiency for parastomal hernia repair in taking the surgeon from ‘incompetent incompetence’ to ‘competent competence’ or mastery.

Further examination of the General Surgical curriculum’s procedure-based requirements (PBA) revealed inadequacies in specifying detailed procedural requirements. The broad classification of ‘hernia repair – all types’ for PBAs neglects critical distinctions in complexity, technique and anatomical considerations. We believe competency assessments should reflect the entire spectrum of hernia procedures, ensuring proficiency in simple procedures and an understanding of complex techniques, which should be performed only by trained and certified complex AWR surgeons.

Limitations of our study include responder bias, likely overestimating training adequacy due to higher response rates among trainees with an interest in abdominal wall surgery. CHERRIES guidelines require that web surveys report the response rate,^5^ i.e. the proportion of respondents to recipients. Therefore, we estimated the number of recipients, trainees and early years consultants we should have reached during questionnaire distribution as 500. However, we could not analyse the characteristics of nonresponders (specialty, deanery, grade, etc) or analyse the extent of responder bias.

However, the high consent rates for logbook verification strengthen the credibility of reported experience. Furthermore, our inclusive range of trainee experience, from senior training years to early consultancy, allows for robust assessment of competency, including the transition to independent practice. Interestingly, although procedural experience increased with seniority, confidence scores did not improve correspondingly, further suggesting the inadequacy of the current training system.

This is the first study to comprehensively assess UK training for abdominal wall surgery, and it reveals critical gaps. We strongly recommend the Joint Committee of Surgical Training (JCST) revise the current curriculum to incorporate procedural guidelines, including defined numbers for a wider range of indicative procedures with established learning curves, and incorporating a clear differentiation between simple and complex hernia repair. Future curriculum changes should give target numbers and competency levels (including PBA targets) for simple hernia procedures for all general surgery trainees. The JCST should then either include evidence-based targets for complex AWR procedures for trainees who select hernia surgery as their specialty of choice or make a post-CCT fellowship mandatory for surgeons hoping to perform complex AWR as part of their practice. Collaboration with AWR experts to develop targeted curricular objectives will better equip future surgeons and enhance patient outcomes after AWR.

## Conflicts

The following authors declare no conflicts of interest: Ruth Willmott; Steve Halligan; Danielle Clyde; Laurie Smith; Priya Daliya; Jonathon Hodgkinson; Thomas Badenoch; Dimitrios Damaskos.

Samuel Parker declares the following conflicts: Bard – speakers fees; Telabio – education grants, speakers fees, research grants; Eurosurgical – research grant.

Dominic Slade declares the following conflicts: LawMed – speakers fees; Cook Biotech – speakers fees; WL Gore Ltd – speakers fees; MedTronic – speakers fees.

David Sanders declares the following conflicts: MedTronic – speakers fees, courses; Intuitive – advisory role; TELA Bio – advisory role.

Jared Torkington declares the following conflicts: MedTronic – on trial steering committee for prophylactic mesh.

Oroog Ali declares the following conflicts: Intuitive – proctor; MedTronic – advisory role.

Rhys Thomas declares the following conflicts: Meshsuture Ltd – speaker fees; TELA Bio – speaker fees, travel grant, faculty fees; BD – travel grant.

## Funding

Steve Halligan is funded by UCLs NIHR Biomedical Research Centre.

The authors received no direct financial support for the research, authorship, and/or publication of this article.
